# A novel prognostic model for transplant-free survival in primary sclerosing cholangitis

**DOI:** 10.1136/gutjnl-2016-313681

**Published:** 2017-07-24

**Authors:** Elisabeth M de Vries, Junfeng Wang, Kate D Williamson, Mariska M Leeflang, Kirsten Boonstra, Rinse K Weersma, Ulrich Beuers, Roger W Chapman, Ronald B Geskus, Cyriel Y Ponsioen

**Affiliations:** 1 Department of Gastroenterology & Hepatology, Academic Medical Center, Amsterdam, The Netherlands; 2 Department of Clinical Epidemiology, Biostatistics and Bioinformatics, Amsterdam Public Health Research Institute, Academic Medical Centre, University of Amsterdam, Amsterdam, The Netherlands; 3 Nuffield Department of Medicine, University of Oxford, Oxford, UK; 4 Translational Gastroenterology Unit, John Radcliffe Hospital, Oxford, Oxfordshire, UK; 5 Department of Gastroenterology and Hepatology, University Medical Center Groningen, Groningen, The Netherlands; 6 Oxford University Clinical Research Unit, Wellcome Trust Major Overseas Programme, Ho Chi Minh City, Vietnam

**Keywords:** Primary Sclerosing Cholangitis

## Abstract

**Objective:**

Most prognostic models for primary sclerosing cholangitis (PSC) are based on patients referred to tertiary care and may not be applicable for the majority of patients with PSC. The aim of this study was to construct and externally validate a novel, broadly applicable prognostic model for transplant-free survival in PSC, based on a large, predominantly population-based cohort using readily available variables.

**Design:**

The derivation cohort consisted of 692 patients with PSC from the Netherlands, the validation cohort of 264 patients with PSC from the UK. Retrospectively, clinical and biochemical variables were collected. We derived the prognostic index from a multivariable Cox regression model in which predictors were selected and parameters were estimated using the least absolute shrinkage and selection operator. The composite end point of PSC-related death and liver transplantation was used. To quantify the models’ predictive value, we calculated the C-statistic as discrimination index and established its calibration accuracy by comparing predicted curves with Kaplan-Meier estimates.

**Results:**

The final model included the variables: PSC subtype, age at PSC diagnosis, albumin, platelets, aspartate aminotransferase, alkaline phosphatase and bilirubin. The C-statistic was 0.68 (95% CI 0.51 to 0.85). Calibration was satisfactory. The model was robust in the sense that the C-statistic did not change when prediction was based on biochemical variables collected at follow-up.

**Conclusion:**

The Amsterdam-Oxford model for PSC showed adequate performance in estimating PSC-related death and/or liver transplant in a predominantly population-based setting. The transplant-free survival probability can be recalculated when updated biochemical values are available.

**Significance of this study**

What is already known on this subject?

- Currently, available prognostic models in primary sclerosing cholangitis (PSC) are based on liver transplant referral centre cohorts, restricting their prognostic value to specific patient groups.
- The most widely used prognostic model in PSC is the Mayo risk score, which is largely based on advanced cases, has a horizon of around 4 years and only predicts death.

What are the new findings?

- The novel Amsterdam-Oxford prognostic model for PSC is based on seven objectively measured and readily available variables: PSC subtype, age at PSC diagnosis, albumin, platelets, aspartate aminotransferase, alkaline phosphatase and bilirubin.
- Long-term transplant-free survival probabilities in PSC can accurately be predicted using the Amsterdam-Oxford model.
- The performance of the Amsterdam-Oxford model remains stable when updated laboratory values are used for prediction in the first few years after diagnosis.

How might it impact on clinical practice in the foreseeable future?

- The novel Amsterdam-Oxford prognostic model for PSC may prove a useful tool for patient counselling and healthcare budget planning.
- In addition, it may be used as a tool for risk stratification in clinical trials, and its prognostic index may even be explored as a candidate surrogate end point.

## Introduction

Primary sclerosing cholangitis (PSC) is an insidious, progressive cholestatic liver disease in which inflammation of the intrahepatic and extrahepatic bile ducts leads to sclerosis, obstruction and destruction of the biliary tract. This results in chronic cholestasis, biliary fibrosis and eventually liver cirrhosis. PSC affects predominantly men (male to female ratio 2:1) at a mean age of 40 years, and up to 70% of patients suffer from concomitant IBD.^1^ PSC disease course is highly variable, with a reported median transplant-free survival from diagnosis onwards, ranging from 13 years in patients seen at tertiary referral centres to 21 years in a population-based cohort.^1^ Various drugs have been studied in PSC, but none has been shown to be efficacious in halting disease progression.^2^ For patients suffering from end-stage liver disease or severe complications of cholestasis, the only curative option is liver transplantation (LTx).^3^

In the absence of medical treatment options for PSC, determining prognosis is important to aid in patient counselling and management, for instance, with regard to optimal patient selection and timing of listing for LTx. The latter is one of the most expensive treatments currently available, hence accurate prediction of future expenditure is of great importance to healthcare officials. Furthermore, an accurate prognostic model may serve as a tool for risk stratification in clinical trials and its prognostic index (PI) may even be explored as a candidate surrogate end point.

Previously, eight prognostic models for PSC have been developed which combined markers for disease progression. (see online supplementary table 1).^4–11^ Most of these models were exclusively based on liver transplant referral centre cohorts, and their predictive value may be restricted to specific patient groups. In addition, definition of end points differed between studies, and only two of these models were externally validated. (see online supplementary table 1).^8 12^

10.1136/gutjnl-2016-313681.supp1 Supplementary Appendix 1

At present, the most widely used prognostic model in PSC is the Mayo risk score, based on the variables age, bilirubin, aspartate aminotransferase (AST), variceal bleeding and albumin.^8^ The time of origin in this study was set at date of referral instead of date at diagnosis, and this model’s prediction is limited to 4 years, and performs best in patients with end-stage PSC.^8^ In addition, the model only estimates time to (all cause) death, and not time to LTx.^8^ Death was projected for those patients that underwent LTx by making an assumption of how long they would have lived had they not undergone LTx. Lastly, 25% of patients were recruited from the placebo and treatment arm of an ursodeoxycholic acid trial, which was justified by virtue of the negative outcome of the trial.^8^ However, ursodeoxycholic acid has an inherent effect on liver biochemistry, hence it may have influenced the prognostic value of the variables in part of the derivation patients.^13–15^

The aim of this study was to construct and externally validate a novel and broadly applicable prognostic model for transplant-free survival in PSC, consisting of readily available disease characteristics and biochemical variables, based on a large, predominantly population-based cohort.

## Methods

### Study design and patients

#### Derivation cohort

The derivation cohort consisted of all patients seen in 44 hospitals that were located in a geographically defined area of six adjacent provinces, comprising 50% of the Dutch population (2007: 7 758 980 inhabitants) (40 basic care centres and 4 academic centres without transplant facilities) and that were alive at January 2000.

Additionally we included 44 patients from a referral centre for LTx outside the geographically defined area. This way we provided a derivation cohort with a case mix that is as representative as possible for most PSC patient series.

#### Validation cohort

The validation cohort included all patients with PSC that visited the John Radcliffe hospital, Oxford, UK, from 1981 onwards. During time of patient inclusion, this centre served as a non-transplant tertiary care liver centre. Patients that did not have follow-up in this hospital after PSC diagnosis were excluded from the validation cohort.

#### Diagnostic inclusion criteria

PSC diagnosis was established according to the European Association for the Study of the Liver guidelines.^16^ Both small and large duct patients were included. A diagnosis of autoimmune hepatitis (AIH) overlap syndrome (PSC-AIH) was made in patients with a characteristic cholangiogram who, in addition, met the simplified AIH criteria.^17^ IBD diagnosis was based on the Lennard-Jones criteria.^18^

### Data collection

Data of clinical and biochemical variables were retrospectively retrieved from patient records. Biochemical parameters that were collected at time of diagnosis (±3 months) included AST, alanine aminotransferase (ALT), alkaline phosphatase (ALP), gamma-glutamyl transpeptidase (γGT), total bilirubin, albumin and platelets. Follow-up liver biochemistry values were collected for AST, ALT, ALP and total bilirubin during the first three years after diagnosis in patients included in the derivation cohort. For patients included in the validation cohort, follow-up liver biochemistry values were collected for all biochemistry variables during the entire length of follow-up.

### Statistical analysis

Patient characteristics were summarised either as median and IQR, or in percentages. As assays may vary between hospitals and over time, biochemical variables were expressed as ratio of upper limit of normal (xULN) or lower limit of normal (xLLN). Since these ratios can only have positive values and were positively skewed, we applied a base 10 logarithmic transformation to all biochemical variables. Missing values were imputed with multivariate imputation by chained equations (MICE),^19^ and 20 imputed data sets were created. (Methods are described in online supplementary appendix.)

The date of PSC diagnosis, defined by the first pathological magnetic resonance cholangiography, endoscopic retrograde cholangiography or liver biopsy, was used as start of follow-up. A composite clinical end point was defined, composed of LTx-related or PSC-related death (death from end-stage liver failure, death from liver surgery, death from cholangiocarcinoma or death from colorectal carcinoma). Data on LTx and death were retrieved from the cohort database.^1^ In case no end point was reached, individuals were censored at the date of last follow-up at the outpatient clinic or—for the derivation cohort—end of the study data collection (January 2012), whichever came first.

All routinely available clinical and biochemical variables that were regarded potentially relevant by expert opinion were assessed as potential predictors. This included age at PSC diagnosis, PSC subtype, sex, IBD type, AIH-overlap syndrome, ursodeoxycholic acid use, AST, ALT, ALP, bilirubin, γGT, albumin and platelets. Since treatment with corticosteroids and other immunosuppressant agents have not demonstrated any improvement in disease activity or in the outcome of PSC, medical therapy options (with the exemption of ursodeoxycholic acid use) were not included as potential predictors.^16 20^

First, the functional form of the relation between the biochemical variables and the risk of the composite end point was investigated in univariate Cox models by means of restricted cubic splines.^21^ Additional variable transformation was performed if strong non-linear effects were shown.

Next, a multivariable Cox regression model was fitted via the least absolute shrinkage and selection operator (Lasso). This penalised likelihood approach creates a model in which several variables are set to zero, whereas others are shrunken to zero to avoid overfitting.^22^ Lasso’s penalty parameter ‘lambda’ was chosen based on the discriminative power of the model, using optimism adjusted Harrell’s C-statistic.^23^ We chose the lambda based on the criterion that the resulting model has as few predictors as possible while still yielding a C-statistic that is no more than 10% below the optimal one. (Methods are described in online supplementary appendix.)

Since we chose a penalty that was larger than the one that gave the highest area under the curve, the parameters may shrink too much. An additional adjustment factor was computed to compensate for this overshrinkage. First, we combined the parameter estimates from the fitted model and the values of the variables to calculate a ‘raw’ PI per individual. The ‘raw’ PI was then used as a single predictor, and the coefficient of this Cox model served as adjustment factor. The final PI was obtained by multiplying the ‘raw’ PI by the adjustment factor. (Methods are described in online supplementary appendix.) The PI only reflects relative hazards but does not give absolute transplant-free survival probability, which is more informative in a clinical setting and is needed for calibration. These can be estimated at any follow-up time if we have an estimate of the baseline ‘survival’.^24^ (Methods are described in online supplementary appendix.)

The model performance was evaluated based on the discriminative power and calibration accuracy in both derivation and validation cohort. The discriminative power is the ability of the model to distinguish high-risk patients from low-risk patients. This was assessed in both the derivation and validation cohort via Harrell’s C-statistic.^23^ The C-statistic was calculated in each of the 20 imputed data sets, and then averaged with Rubin’s rule.^25 26^ For assessing calibration accuracy, we divided patients into four risk groups, based on their PIs, using threshold points at 16th, 50th and 84th percentiles in the derivation data.^24^ Next, we compared the averaged predicted survival probability with the observed Kaplan-Meier survival probability in the four risk groups. For the validation cohort, we recalibrated the baseline survival before estimating the probability of survival for each individual. (Methods are described in online supplementary appendix.)

We also assessed whether the discriminative power of the model remained stable over time by calculating the C-statistic using updated data at 1, 2 and 3 years after diagnosis.

Statistical analyses were performed using R V.3.1.2,^27^ and packages MICE, rms, glmnet and ggplot2. The reporting of this prognostic model study followed the recommendations in Steyerberg *et al* and Transparent Reporting of a multivariable prediction model for Individual Prognosis Or Diagnosis (TRIPOD) statement (TRIPOD checklist can be found in the online supplementary appendix).^28 29^

## Results

### Patient characteristics

The derivation cohort included 692 patients of which 447 (65%) were male, and 630 (91%) were diagnosed with large duct PSC. The median age at PSC diagnosis was 37 years (IQR 27–49). The median follow-up time was 110 months (IQR 69–184) (table 1).

**Table 1:** Patient characteristics at diagnosis

	Derivation cohort (n=692)	Missing values (n (%))	Validation cohort (n=264)	Missing values (n (%))
Male (n (%))	447 (65)		165 (63)	
Large duct PSC (n (%))	630 (91)		231 (88)	
Age at diagnosis PSC (years) (median (IQR))	37 (27–49)		45 (33–57)	
AIH overlap (n (%))	37 (5)		5 (2)	
IBD (n (%))	480 (70)		195 (74)	
UC (n (%))	373 (54)		143 (54)	
Crohn’s disease (n (%))	89 (13)		39 (15)	
Unspecified (n (%))	18 (3)		13 (5)	
Ursodeoxycholic acid (n (%))*	326 (80)	281 (41)		
Follow-up time (months) (median (IQR))	110 (69–184)		103 (53–153)	
PSC-related death (n (%))	71 (10)		37 (14)	
Liver transplantation (n (%))	121 (18)		18 (7)	
Alkaline phosphatase (xULN) (median (IQR))	1.97 (1.43–3.28)	255 (37)	2.27 (1.27–3.83)	177 (67)
Aspartate aminotransferase (xULN) (median (IQR))	1.55 (1.0–3.03)	169 (24)	1.21 (0.79–2.05)	198 (75)
Alanine aminotransferase (xULN) (median (IQR))	2.29 (1.34–4.66)	268 (39)	1.40 (0.88–2.58)	238 (90)
Bilirubin (xULN) (median (IQR))	0.82 (0.53–1.71)	208 (30)	0.71 (0.47–1.29)	178 (67)
Gamma-glutamyl transpeptidase (xULN) (median (IQR))	6.11 (3.36–11.88)	282 (41)	7.15 (1.98–14.30)	210 (80)
Albumin (xLLN) (median (IQR))	1.14 (1.02–1.23)	312 (45)	1.23 (1.11–1.31)	182 (69)
Platelets (xLLN) (median (IQR))	1.79 (1.36–2.29)	375 (54)	1.89 (1.53–2.75)	189 (71)
AIH, autoimmune hepatitis; PSC, primary sclerosing cholangitis; xLLN, lower limit of normal; xULN, upper limit of normal.				

The validation cohort consisted of 264 patients, of which 165 (63%) were male. A total of 231 (88%) patients had large duct PSC and the median age at PSC diagnosis was 45 (IQR 33–57) years. The median follow-up time was 103 months (IQR 53–153) (table 1).

**Figure 1:**
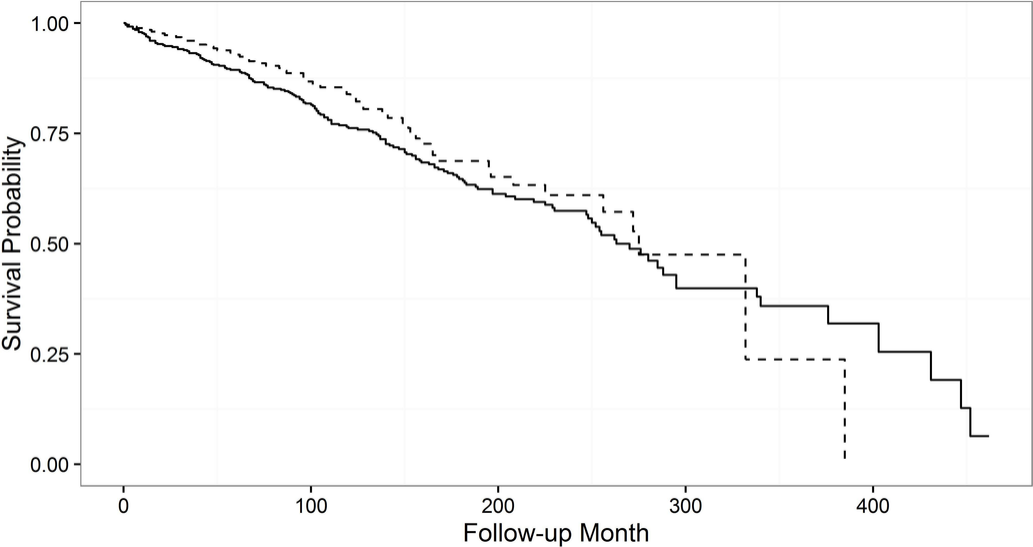
Kaplan-Meier curves for derivation and validation cohorts. Solid line: derivation cohort (the Netherlands); dashed line: validation cohort (the UK).

The transplant-free survival probability of patients included in the derivation cohort was slightly lower compared with the validation cohort (figure 1).

Laboratory values represent measured values at time of diagnosis (±3 months), missing values represent the amount of missing data at time of diagnosis (±3 months). Follow-up laboratory values were available for most individuals (not depicted in this table) and used to impute laboratory values at time of diagnosis. Imputed (laboratory) data were used for model development.

### Prognostic model development

#### Coding of predictors

For categorical predictors (PSC subtype, sex, IBD type, AIH-overlap syndrome, ursodeoxycholic acid use), dummy variables were created.

It was observed that the relationship between platelets and outcome followed a U-shaped pattern, with a turning point around 3.16xLLN. Therefore, the values for this variable were transformed by taking the absolute distance between log(platelets) (in unit xLLN), and the turning point log(3.16)≈0.5. No strong non-linear effect was observed in other biochemical variables. The detailed coding of all predictors can be found in online supplementary table 2; an example is given in the online supplementary appendix.

#### Multivariable analysis and model selection

The model with the highest C-statistic included a total of 13 variables. A model including 13 variables would not be easy to use in clinical practice. When allowing for a performance of 10% below the optimal C-statistic, seven variables remained in the model: PSC subtype, age at PSC diagnosis, ALP, AST, bilirubin, platelets and albumin (see online supplementary figure 1).

#### Calculation of the PI

The final PI was calculated as 1.890 times the ‘raw’ PI and is structured as

PI=0.323*PSC subtype +0.018*Age at diagnosis – 2.485*Albumin+2.451*Platelets+0.347*AST+0.393*ALP+0.337*Total Bilirubin (Formula 1)

- PSC subtype: large duct PSC=1; small duct PSC=0
- AST, ALP and Total Bilirubin are expressed in xULN, transformed to (10–log)
- Albumin is expressed in xLLN, transformed to (10–log)
- Platelets are expressed in xLLN, transformed to abs(10–log–0.5)

**Figure 2:**
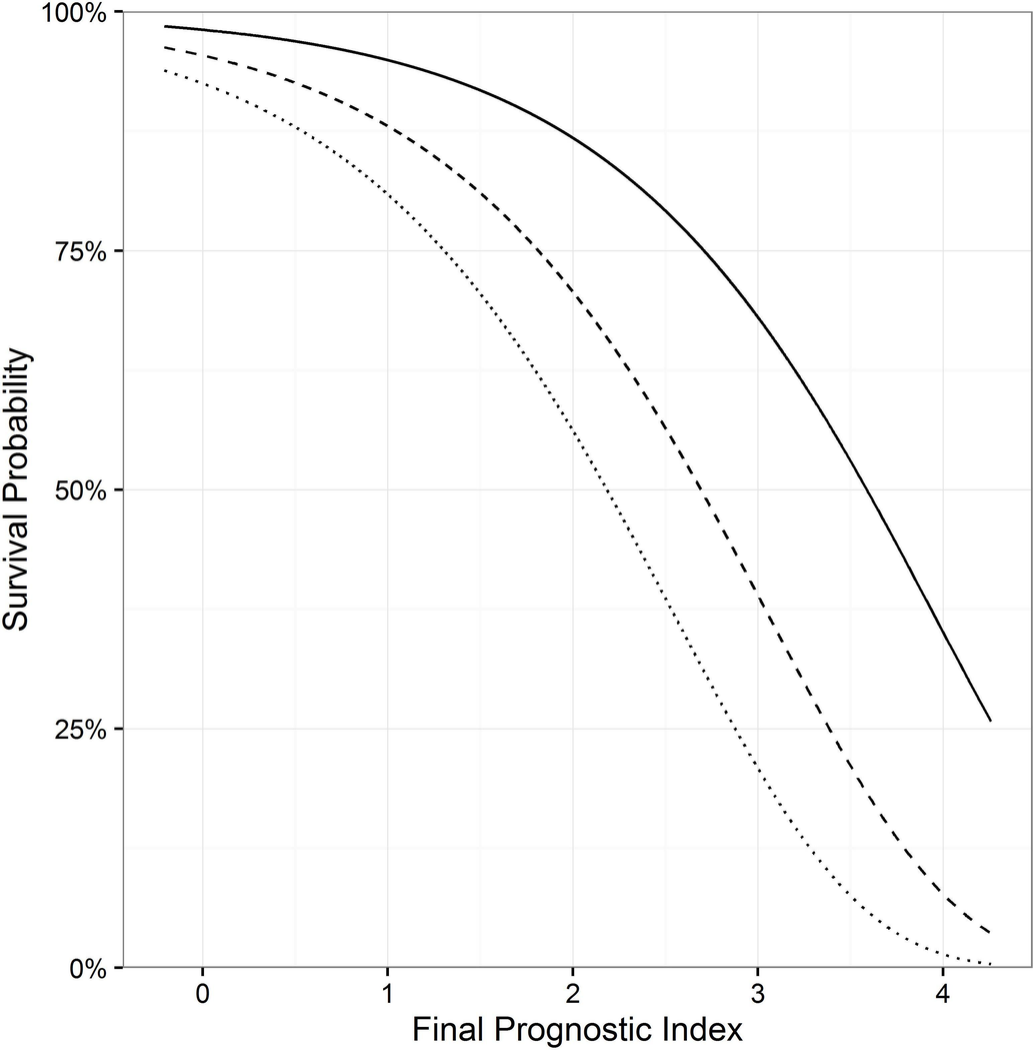
Prediction of 5-year, 10-year and 15-year survival probability versus final prognostic index. Solid line: 5 years; dashed line:10 years; dotted line: 15 years.

The distribution of the final PI is plotted in the upper half of online supplementary figure 2. In figure 2, the relation between the PI and 5-year, 10-year and 15-year survival probabilities is illustrated. To calculate the survival probability for an individual patient at any year after diagnosis, the baseline survival probabilities are provided in online supplementary table 3. An online calculator can be accessed for this calculation: http://www.amc.nl/psc Furthermore, a patient example of how the model can be used in clinical practice can be found in the online supplementary appendix.

### Evaluation of model performance

#### Discriminative power

The discriminative power was used as one criterion in the variable selection during the model development, using the derivation cohort, and will therefore not be evaluated again in the same cohort.

To assess the discriminative power of the model in the validation cohort, the PI of each individual included in the validation cohort was calculated using Formula 1. The distribution of the final PI in the validation cohort is plotted in online supplementary figure 2. The discriminative power of the model in the validation cohort was 0.68 (95% CI 0.51 to 0.85).

#### Calibration accuracy

For the derivation cohort, the observed Kaplan-Meier survival curves of the four risk groups that we defined (see online supplementary table 4) were all close to the mean predicted survival curves, indicating good calibration (see online supplementary figure 3A). In addition, these curves were well separated, which confirmed the discriminative power of the PI (see online supplementary figure 3A).

To assess the calibration accuracy of the prognostic model in the validation cohort, we classified the patients into four risk groups based on the same thresholds as used in the derivation cohort. The thresholds and proportion of patients included in each risk group are shown in online supplementary table 4. The mean predicted survival curves were comparable with the observed Kaplan-Meier curves per risk group (see online supplementary figure 3B). And the Kaplan-Meier survival curves of the risk groups were well separated, confirming the high discriminative power of the PI in the validation cohort.

#### Performance of the model over time

The C-statistics based on data at 1, 2 and 3 years of follow-up were very comparable in the validation cohort (see online supplementary table 5).

## Discussion

This study provides a novel prognostic model for transplant-free survival of patients with PSC, based on a unique, well-phenotyped, predominantly population-based PSC cohort. The model is based on seven objectively measured and readily available variables: PSC subtype, age at PSC diagnosis, ALP, AST, total bilirubin, albumin and platelets. Validation of the model in an external PSC cohort showed its robustness and adequate performance.

Recently, results of a joint effort from the International PSC Study Group, studying clinical risk factors for disease course cohort comprising 7121 cases, were published.^30^ In addition to PSC subtype and age at PSC diagnosis, sex and IBD subtype were also associated with disease course. This is in line with the results of our study since we also found that both sex and IBD subtype were predictive factors for the composite end point LTx or PSC-related death. For practical reasons of computing a prognostic model, we applied a threshold allowing for <10% reduction in C-statistic, and so sex and IBD subtype were not chosen as one of the seven most prognostic parameters. (see online supplementary figure 1). In the present study, the parameters sex and IBD subtype played a less important prognostic role as compared with the International PSC Study Group (IPSCSG) cohort, which might be explained by the fact that our derivation and validation cohorts were mostly population-based, while most of the other cohorts that comprised the International PSC Study Group cohort were tertiary referral cohorts. Another factor which may explain this difference is that the composite end point used in the International PSC Study Group cohort was a combination of LTx and all-cause mortality, instead of PSC-related mortality in our study. Of note, our definition included death from colorectal cancer, the risk of which is clearly elevated in patients with PSC/UC.

The usefulness and applicability of previously composed prognostic models has been hampered by the use of tertiary referral-only cohorts, and variation in definitions of the time of origin in the studies (date of PSC diagnosis vs date of referral), PSC diagnosis and clinical end points (see online supplementary table 1). By using a predominantly population-based cohort as the derivation cohort, the present study largely mitigated these limitations. In addition, established definitions for PSC diagnosis and clinical end points were used, creating a prognostic model that represents a heterogeneous population of patients with PSC and is broadly applicable.^16^

The optimal way to validate a prognostic model is to assess its performance in an independent, but similar patient cohort.^24^ We used a PSC patient cohort from the UK. A notable difference in frequency of LTx between the derivation and validation cohort was observed. This may in part have resulted from the non-population-based 44 patients included in the derivation cohort, who were retrieved from a referral transplant centre. This may have led to a relative over-representation of more severely diseased patients with PSC. Thus the model may overestimate the individual’s risk of reaching the end point when applied to patients in other settings. However, including a case mix of both population-based and referral cohort PSC patients in the derivation cohort, we believe, gives the optimal representation of most PSC patient series.

The biochemistry parameters that are included in the model will in general change throughout the disease course. Early biochemical markers include an elevated serum ALP—the hallmark of PSC—and serum AST and ALT levels that are often elevated up to 2–3 xULN at time of diagnosis.^16^ In contrast, serum total bilirubin is often normal at PSC diagnosis and will increase in case of advanced disease stage, a dominant stricture or suppurative cholangitis. Serum albumin will only decrease once hepatic synthetic function is affected in advanced disease. The same holds true for serum platelets, which will only deviate from normal in advanced disease stage. The liver biochemistry variables implemented in the model are measured at every routine patient check, thus are frequently updated, objective, and readily available. This greatly benefits the applicability of the present model in clinical practice.

In a recent study assessing the prognostic value of ALP, it was demonstrated that ALP levels 1 year after diagnosis (T1) had a better prognostic value compared with ALP values at diagnosis or percentage change between diagnosis and T1.^31^ Given this result, we also made an attempt to develop another model based on T1 data, following the same modelling strategy. Compared with the current model, this T1 model included two more variables and yielded a lower C-statistic.

To assess if the current model—based on data at time of diagnosis—was also applicable at later time points, additional C-statistics were calculated when using laboratory values at a follow-up of 1, 2 and 3 years. Similar C-statistics were found, indicating that the model has good performance also when it is used for prediction at the first years after diagnosis. This suggests that the effects of interventions after diagnosis is made, such as endoscopic treatment of dominant strictures, may not have a sizeable effect on the performance of the model.

The calibration accuracy in the validation cohort was acceptable after recalibrating the baseline survival probability, represented by the difference in mean predicted transplant-free survival curves, when compared with the observed Kaplan-Meier curves per risk group. This indicates that recalibration should be considered when the model is applied to a different cohort. A prognostic model developed based on patients from one country may not always be valid for patients from other parts of the world.^32^ With this study, we have reported all the information that is necessary for further recalibration, or revision of the current model, to fit a specific external patient cohort.

Current epidemiological data on the natural history of PSC—including our Epi PSC PBC cohort—are predominantly based on Western, mostly Caucasian populations, and cohorts including children are lacking. However, there seems to be a geographical variation in incidence and prevalence rates, with lower rates in populations from Southern European and Asian descent.^33^ Unfortunately, true population-based studies are scarce, and none has been performed in Asia and Africa.^33^ Factors that are suggested to play a role in the variable global distribution of PSC are differences in frequency of IBD, and in Human Leukocyte Antigen (HLA) susceptibility between ethnic populations.^34–37^ To be able to confirm the applicability of the present prognostic model in all populations, a better understanding of possible differences in incidence, prevalence and natural history between various ethnic and racial populations, and age groups is warranted.

Because variceal bleeding was not recorded for most patients, we were not able to compare the performance of the present model with the Mayo risk score. Contrary to the Mayo risk score, which has a horizon of only 4 years, the present model includes a more than three times longer follow-up time and was based on patients retrieved from a predominantly population-based cohort. Hence, we believe our model has a broader applicability compared with the Mayo risk score. Retrospective data collection always has the inherent drawback of incomplete data collection. Consequently, biochemical values measured at time of diagnosis were missing for a considerable amount of cases. Most missing data were caused by patients that were diagnosed a long time ago as a result of which their patient files were no longer accessible. Therefore, these missing data were considered to be missing at random since they were only related to the year of diagnosis which can be observed and was included in the imputation model. Multiple imputation with two-level linear model enabled us to impute these biochemical values using data collected during follow-up from the same individual, thereby improving the validity of the results.^38^

In conclusion, with this novel Amsterdam-Oxford prognostic model for PSC consisting of seven clinical and biochemical variables, long-term transplant-free survival probabilities of patients with PSC can accurately be predicted. It may prove a useful tool for patient counselling, healthcare budget planning, as well as for risk stratification in clinical trials.
