# Supplementary material for: A novel prognostic model for transplant-free survival in primary sclerosing cholangitis
Source: Gut. 2017 Jul 24;67(10):1864–9. doi: 10.1136/gutjnl-2016-313681 (PMC6145288; doi:10.1136/gutjnl-2016-313681)
Supplement: Supplementary Appendix 1 [file gutjnl-2016-313681supp001.pdf]

Supplementary Appendix

Table of Contents Page

1 Statistical Methods in Model Derivation and Validation

1.1 Multiple imputation of missing values ……………………………………….....2-3

1.2 Variable selection by Lasso ……………………………………………………….3-4

1.3 Adjustment factor through recalibration …………………………………........4-5

1.4 Estimate Baseline Survival and Individual Survival ……………………………….5

1.5 Comparing predicted and observed survival in risk groups………………………...5

2 Model use in clinical practice …………………………………………………….6-7

REFERENCES……………………………………………………………………….8

Supplementary Figure 1………………………………………………………………..9

Supplementary Figure 2……………………………………………………………….10

Supplementary Figure 3……………………………………………………………….11

Supplementary Table 1……………………………………………………………...12-13

Supplementary Table 2………………………………………………………………..14

Supplementary Table 3………………………………………………………………..15

Supplementary Table 4………………………………………………………………..16

Supplementary Table 5………………………………………………………………..17

TRIPOD Checklist: Prediction Model Development and Validation……………….18-19

1 Statistical Methods in Model Derivation and Validation

1.1 Multiple imputation of missing values

Biochemical variables (aspartate aminotransferase (AST), alanine aminotransferase (ALT), alkaline phosphatase (ALP), gamma-glutamyl transpeptidase (γGT), total bilirubin, albumin and platelets) were measured before diagnosis, at time of diagnosis, and during follow-up. In table 1, we considered values that were obtained within three months (+/- 3 months) from diagnosis as baseline values. The percentage of missing values (i.e. no value within 3 months from diagnosis) can be found in table 1 as well.

Biochemical variables were expressed as ratio of upper limit of normal (xULN) or lower limit of normal (xLLN) and transformed with a base 10 logarithmic transformation before the imputation.

Missing values were imputed using the multivariable imputation by chained equations (MICE) technique. We created 20 imputed datasets. In each imputation, the number of iterations was set at 25.

For continuous time-fixed variables predictive mean matching was used; for binary variables logistic regression was used; for variables that could change over time (AST, ALT, ALP and total bilirubin) a two-level linear model (2l.norm in the mice R package) was used and we used the predicted value at T0 as baseline value in our model.

Although MICE is a powerful approach to address missing data, we are also aware of the limitations of MICE.[1] As with most imputation methods, it assumes missing at random (MAR). Since the missing biochemical data at diagnosis were only related to the year of diagnosis, in this study, the missing biochemical data at diagnosis can be

considered to be missing at random. Also, an imputation model can be mis-specified, which may bias the estimates of partially missing variables. To prevent this, we also included the outcome variable in the imputation model. The limitations and pitfalls of MICE were extensively discussed by White et al.[1]

1.2 Variable selection by Lasso

The potential problems when using Lasso with multiple imputed datasets and the potential solutions are discussed by Luijk et al.[2] The selected variables may differ per imputed data set, which is problematic when we want to average over the parameter estimates from the 20 imputed data sets. Therefore, we stacked all data sets to create a long combined dataset.[3]

Due to the fact that Lasso penalizes single parameter estimates, one variable can only have one single parameter in the model. Therefore, we used restricted cubic splines in univariable Cox models to investigate whether the relation between the (transformed) biochemical variables and the risk of the composite endpoint could be assumed linear.. To avoid complexity, additional variable transformation was performed only if strong non-linear effects were shown. Lasso's penalty parameter 'lambda' was chosen based on the discriminative power of the model, using Harrell's C-statistic.[4] The C-statistic was corrected for optimism using the bootstrap procedure (1000 resamples).[5] In each generated bootstrap, sample patients were selected not rows, meaning that if one patient was selected in the sample, the data of this patient will be selected in all 20 imputations.

We chose the lambda based on the criterion that the resulting model has as few predictors as possible while still yielding a C-statistic that is not more than 10% below the optimal one. Within a range of lambda's with the same number of predictors, we chose the one that had the highest C-statistic.

1.3 Adjustment factor through recalibration

Since the penalty parameter lambda was chosen larger than optimal for reasons of parsimony, the parameters may shrink too much, which impairs calibration. For this reason an additional adjustment factor was computed to compensate for over-shrinkage.

First, the "raw" PI was calculated based on the Lasso model (described in paragraph 1.2). In the recalibration step, the raw PI was used as a single predictor in a Cox model on the same dataset, and the coefficient of this Cox model served as the adjustment factor. In other words, as the final PI we chose the "raw" PI multiplied by the adjustment factor.

There is a risk that this adjustment factor will inflate the parameters too much. However, the relation between the parameters cannot change (they were all multiplied by the same adjustment factor), and therefore the risk of over-fitting is minimized.

The recalibration also allowed us to correct for left truncation caused by the fact that for some individuals the diagnosis was made before the start of the follow-up. We needed to correct for the fact that individuals that died before 2000 could not enter the study. Left truncation adjustment was needed only for the derivation cohort. Patients diagnosed before the year 2000 were entered into the analysis at 2000, and we excluded patients that received a liver transplant before 2000.

1.4 Estimate Baseline Survival and Individual Survival

Based on the model with the final PI, the baseline time-to-event ("survival" curve $S_0(t)$) was estimated. This corresponds to the time-to-event distribution of an "average" patient, i.e. a person with the average value of the PI in the derivation cohort. Then the "survival" curve of other patients can be computed via the formula: $S_i(t) = S_0(t)^{Exp(PI_i - \overline{PI})}$, where PI_i is the prognostic index of patient i and $\overline{\mathrm{PI}}$ is the average PI in derivation cohort, Exp stands for the exponential function.

1.5 Comparing predicted and observed survival in risk groups

To assess the calibration accuracy of the validation cohort, we classified the patients into four risk groups, and then we compared the mean predicted survival curves with the observed Kaplan-Meier curves per risk group. To get the mean predicted survival curve for each risk group, we first calculated the survival estimates for each individual by using the formula described in paragraph 1.4; then for each follow-up time t, we averaged the survival estimates from all the individuals in the same risk group.

2 Model use in clinical practice

The following example explains how the model can be used in clinical practice:

Suppose a forty year old male patient visits the outpatient clinic. He was recently diagnosed with large duct PSC, and his blood tests show the following results:

- ☐ *Albumin (g/L) 38 (reference value (LLN): 35)*
- ☐ *Platelets: 250 x 10^9/L (reference value (LLN): 150 x 10^9/L)*
- ☐ *AST (U/L): 88 (reference value (ULN): 35)*
- ☐ *Alkaline phosphatase (U/L): 360 (reference value (ULN): 120)*
- ☐ *Bilirubin (umol/L): 26 (reference value (ULN): 17)*

When we transform clinical and laboratory data according to the methods described in paragraph 1.1 we get the following numbers to be entered into formula 1

(Step 1: expression in xULN/xLLN; Step 2: variable transformation):

- ☐ PSC subtype large duct: 1
- ☐ Age at diagnosis: 40
- ☐ Albumin (g/L)
    - o Step 1: 38/35 = 1.1xLLN; Step 2: log(1.1) = 0.041
- ☐ Platelets
    - o Step 1: 250/150=1.667xLLN; Step 2: abs(log(1.667)-0.5) = 0.270
- ☐ AST (U/L)
    - o Step 1: 88/35 = 2.514xULN; Step 2: log (2.514) = 0.398
- ☐ Alkaline phosphatase (U/L):
    - o Step 1: 360/120 = 3.0xULN; Step 2: log(3.0) = 0.477
- ☐ Bilirubin (umol/L)

- Step 1: 26/17= 1.529xULN; Step 2: log (1.529) = 0.176

When entering these transformed data into the formula one can calculate the prognostic index:

PI = 0.323*1 + 0.018*40 - 2.485*0.041 + 2.451*0.270 + 0.347*0.398 + 0.393*0.477 + 0.337*0.176 = 1.986

To calculate the probability to remain event-free for 5, 10 and 15 years, the user needs the baseline "survival" estimate - in Supplementary Table 3 this is given for each year-, as well as the mean of the final PI's in the derivation cohort, which is 1.646.

Then we obtain 5-year probability:

$$\hat{S}_i(5) = \hat{S}_0(5)^{\exp\,(PI_i - \overline{PI})} = 0.912^{\exp\,(1.986-1.646)} = 87.86\%$$

10-year probability:

$$\hat{S}_i(10) = \hat{S}_0(10)^{\exp\,(PI_i - \overline{PI})} = 0.789^{\exp\,(1.986-1.646)} = 71.68\%$$

15-year probability:

$$\hat{S}_i(15) = \hat{S}_0(15)^{\exp\,(PI_i - \overline{PI})} = 0.674^{\exp\,(1.986-1.646)} = 57.45\%$$

The relation between final PI and the probability to remain event-free is illustrated in Figure 2.

REFERENCES

1 White IR, Wood AM. Multiple imputation using chained equations : Issues and guidance for practice. *Stat Med* 2011;:377–99.

2 Luijk R. The group lasso in the proportional hazards model with an application to multiply imputed high-dimensional data. Master's thesis. *Leiden Univ* 2012;:https://www.math.leidenuniv.nl/en/theses/305/.

3 Wan Y, Datta S, Conklin DJ, *et al.* Variable selection models based on multiple imputation with an application for predicting median effective dose and maximum effect. *J Stat Comput Simul* 2015;**85**:1902–1916.

4 Harrell FJ, Lee K, Mark D. Multivariable prognostic models: issues in developing models, evaluating assumptions and adequacy, and measuring and reducing errors. *Stat Med* 1996;**15**:361–87.

5 Harrell, F. E. J. *Regression Modeling Strategies: With Applications to Linear Models, Logistic Regression, and Survival Analysis*. 2001.

Supplementary Figure 1. Model selection procedure by tuning lambda.

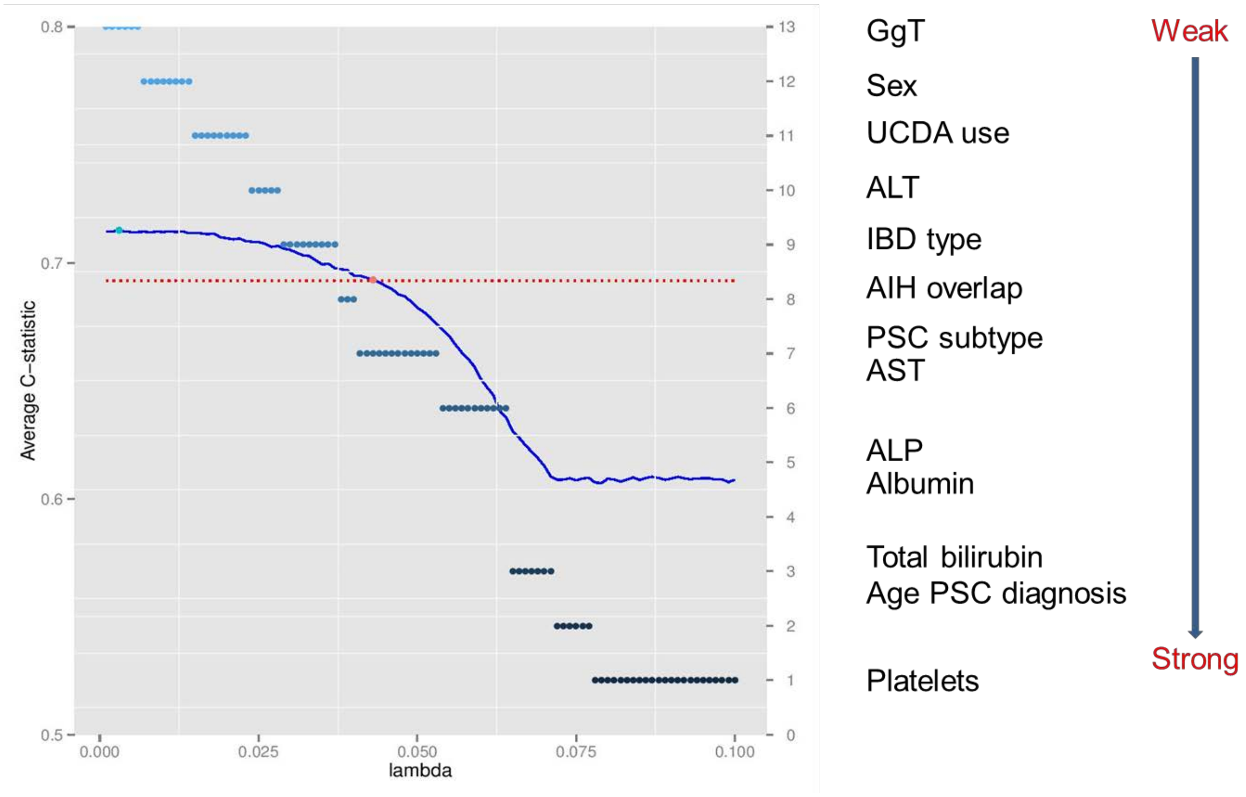

X axis: Lasso’s penalty parameter ‘lambda’, ranges from 0 - 0.1.
Left Y axis: Harrell's C-statistic after adjustment for optimism.
Right Y axis: Number of variables in the model.
The blue line corresponds to the left Y axis, which represents the trend of the C-statistic as lambda increases.
The horizontal red line represents the minimal performance requirement, which is 10% below the optimal performance for our model.
Where the blue line crosses the red horizontal line is the largest lambda with which the model can still meet the performance requirement.
From there the red vertical line is drawn to show at that lambda value, 7 variables were selected.
The yellow vertical line represents the optimal performance we could achieve with these 7 variables, and the final lambda that was chosen (0.041).

Supplementary Figure 2. Distribution of prognostic index in the derivation and validation cohorts. Upper part: derivation cohort; lower part: validation cohort.

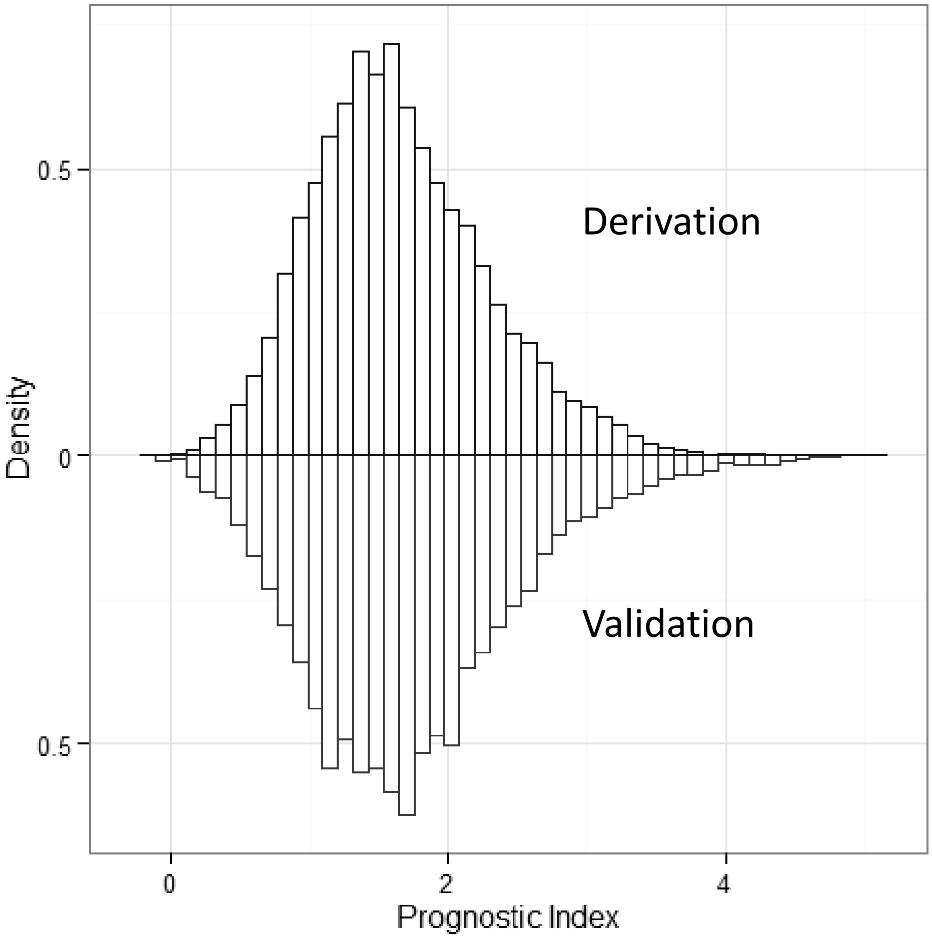

Supplementary Figure 3. Predicted versus observed survival probability per risk group (1-4)
(A) Derivation cohort (B) Validation cohort (baseline hazard recalibrated).
Solid line: Observed Kaplan-Meier Curve; Dashed line: Mean predicted survival curves.

A

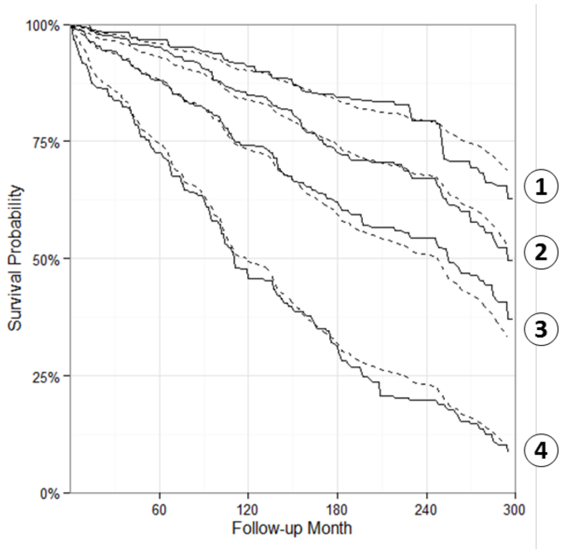

B

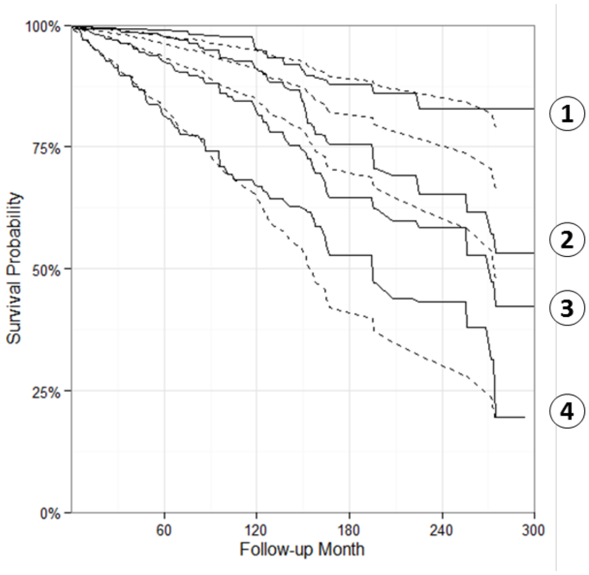

Supplementary Table 1. Previous prognostic models for primary sclerosing cholangitis

**First Author**	**Year**	**Patient cohort: FU, survival time**	**Endpoint**	**Variables included in the prognostic model**											
				Age	Histo logy	Hepatomegaly	Splenomegaly	Variceal bleeding	IBD	ALP	Bilrubin	AST	Albumin	Hb	Intra-extrahep cholangiogram
**Wiesner et al.**	1989	N=174. Mayo clinic's cohort,: referral center US<br>FU: mean 6yr (range 2.7-15.5)<br>Survival: median 11.9 yrs	-Death, all cause<br>- LTx	X	X				X		X			X	
**Farrant et al.**	1991	N=126. Kings College; referral center UK<br>FU: median 5.8 yrs,<br>Survival: medium 12 yrs	-Death, from liver related disease<br>-LTx	X	X	X	X			X					
**Dickson et al.**	1992	N=426. Five medical referral centers<br>UK and US<br>FU: median 3 yrs (range, 0.01-16.6 years)<br>Survival: not described	Death, all cause	X	X		X				X				
**Broome et al.**	1996	N=305. Nine 9 Swedish centers, all served as primary and referral centers<br>FU: median 63 months (range 1-194)<br>Survival: medium 12 yrs	-Death, from liver related disease<br>-LTx	X	X						X				
**Kim et al.**	2000	N=529. Patients included in Dickson trial + patient from UDCA study Mayo, US.<br>N=405 model building,<br>N=124 validation (patients from Kings College)<br>FU: median 3 3 yrs (range 0.01-25,1)<br>Survival: not described	-Death, all cause	X				X			X	X	X		

**Boberg et al.**	2002	N=330. Five European centers, all served as primary and referral centers<br>FU: median 8.4 yrs<br>Survival: median 11.7 yrs	-Death, from PSC<br>-LTx	X							X		X		
**Ponsioen et al.**	2002<br><br><br><br>2010	N=130. Four Dutch centers,1 referral, 3 primary centers<br>FU: median 4 yrs (0.2-18.4)<br>Survival: median 18 yrs<br>N= 111. One center Olso, referral center<br>FU: median 6.3 yrs (range 0.1-26.2)	-Death, from liver disease<br>-LTx<br><br>-Death, from liver disease<br>-LTx	X											X<br><br><br><br>X
**Tischendorf et al.**	2007	N=273.medical school of Hannover, Germany, referral center.<br>FU: median 6.3 yrs (range 0.1-23.3)<br>Survival: median 9.3 yrs	- Death, all cause<br>- LTx	X		X	X				X		X		X

Supplementary Table 2. Coding of predictors

Variable	Coding (x stands for original value)
age at PSC diagnosis	=x
PSC subtype	Dummy (1=Large duct, 0=Small duct)
sex	Dummy (1=Female, 0=Male)
IBD type	Dummy (1=Yes, 0=No)
AIH-overlap syndrome	Dummy (1=Yes, 0=No)
ursodeoxycholic acid use	Dummy (1=Yes, 0=No)
AST	=log10(x/ULN)
ALT	=log10(x/ULN)
ALP	=log10(x/ULN)
bilirubin	=log10(x/ULN)
γ GT	=log10(x/ULN)
albumin	=log10(x/LLN)
platelet	=abs(log10(x/LLN)-0.5)

Supplementary Table 3. Baseline survival probability of the prognostic model.

Year	Baseline Survival	Year	Baseline Survival
0	1	13	0.718900081
1	0.976003276	14	0.695637963
2	0.958190391	15	0.674046812
3	0.944872387	16	0.649928168
4	0.921801174	17	0.632527844
5	0.911738594	18	0.626239076
6	0.886368701	19	0.613255379
7	0.870414839	20	0.599392599
8	0.844120125	21	0.564462416
9	0.812447482	22	0.527004006
10	0.788752707	23	0.503629598
11	0.781935324	24	0.456791571
12	0.745666457	25	0.425503951

Supplementary Table 4. Thresholds and corresponding proportion of patients included in risk groups 1-4 .

Risk Group	Range in PI	Proportion	
		Derivation	Validation
**1. Low risk**	<1.032	16.0%	16.7%
**2. Low- Intermediate risk**	1.032 - 1.578	34.0%	28.8%
**3. Moderate risk**	1.578 - 2.266	34.0%	33.5%
**4. High risk**	>=2.266	16.0%	21.0%

Supplementary Table 5. C-statistics of PI calculated at n years of follow-up.

	Derivation Cohort		Validation Cohort	
**Follow-up**	C-statistic	95% CI	C-statistic	95% CI
**1 year**	0.681	(0.588, 0.775)	0.664	(0.604, 0.724)
**2 years**	0.686	(0.590, 0.781)	0.657	(0.594, 0.721)
**3 years**	0.665	(0.564, 0.767)	0.664	(0.598, 0.729)

TRIPOD Checklist: Prediction Model Development and Validation

Section/Topic			Checklist Item	Page
**Title and abstract**				
Title	1	D;V	Identify the study as developing and/or validating a multivariable prediction model, the target population, and the outcome to be predicted.	1
Abstract	2	D;V	Provide a summary of objectives, study design, setting, participants, sample size, predictors, outcome, statistical analysis, results, and conclusions.	4
**Introduction**				
Background and objectives	3a	D;V	Explain the medical context (including whether diagnostic or prognostic) and rationale for developing or validating the multivariable prediction model, including references to existing models.	5
	3b	D;V	Specify the objectives, including whether the study describes the development or validation of the model or both.	6
**Methods**				
Source of data	4a	D;V	Describe the study design or source of data (e.g., randomized trial, cohort, or registry data), separately for the development and validation data sets, if applicable.	8
	4b	D;V	Specify the key study dates, including start of accrual; end of accrual; and, if applicable, end of follow-up.	8
Participants	5a	D;V	Specify key elements of the study setting (e.g., primary care, secondary care, general population) including number and location of centres.	8
	5b	D;V	Describe eligibility criteria for participants.	8
	5c	D;V	Give details of treatments received, if relevant.	NA
Outcome	6a	D;V	Clearly define the outcome that is predicted by the prediction model, including how and when assessed.	10
	6b	D;V	Report any actions to blind assessment of the outcome to be predicted.	NA
Predictors	7a	D;V	Clearly define all predictors used in developing or validating the multivariable prediction model, including how and when they were measured.	9
	7b	D;V	Report any actions to blind assessment of predictors for the outcome and other predictors.	NA
Sample size	8	D;V	Explain how the study size was arrived at.	13
Missing data	9	D;V	Describe how missing data were handled (e.g., complete-case analysis, single imputation, multiple imputation) with details of any imputation method.	9
Statistical analysis methods	10a	D	Describe how predictors were handled in the analyses.	13
	10b	D	Specify type of model, all model-building procedures (including any predictor selection), and method for internal validation.	10-11
	10c	V	For validation, describe how the predictions were calculated.	17
	10d	D;V	Specify all measures used to assess model performance and, if relevant, to compare multiple models.	11,16-17
	10e	V	Describe any model updating (e.g., recalibration) arising from the validation, if done.	12
Risk groups	11	D;V	Provide details on how risk groups were created, if done.	11
Development vs. validation	12	V	For validation, identify any differences from the development data in setting, eligibility criteria, outcome, and predictors.	13
**Results**				
Participants	13a	D;V	Describe the flow of participants through the study, including the number of participants with and without the outcome and, if applicable, a summary of the follow-up time. A diagram may be helpful.	13
	13b	D;V	Describe the characteristics of the participants (basic demographics, clinical features, available predictors), including the number of participants with missing data for predictors and outcome.	15
	13c	V	For validation, show a comparison with the development data of the distribution of important variables (demographics, predictors and outcome).	14
Model development	14a	D	Specify the number of participants and outcome events in each analysis.	13
	14b	D	If done, report the unadjusted association between each candidate predictor and outcome.	NA
Model specification	15a	D	Present the full prediction model to allow predictions for individuals (i.e., all regression coefficients, and model intercept or baseline survival at a given time point).	16
	15b	D	Explain how to the use the prediction model.	Appendix
Model performance	16	D;V	Report performance measures (with CIs) for the prediction model.	17
Model-updating	17	V	If done, report the results from any model updating (i.e., model specification, model performance).	17
**Discussion**				
Limitations	18	D;V	Discuss any limitations of the study (such as nonrepresentative sample, few events per predictor, missing data).	19-20

Interpretation	.9a	V	For validation, discuss the results with reference to performance in the development data, and any other validation data.	16-17
	.9b	);V	Give an overall interpretation of the results, considering objectives, limitations, results from similar studies, and other relevant evidence.	19-20
Implications	20	);V	Discuss the potential clinical use of the model and implications for future research.	21
**Other information**				
Supplementary information	21	);V	Provide information about the availability of supplementary resources, such as study protocol, Web calculator, and data sets.	Appendix
Funding	22	);V	Give the source of funding and the role of the funders for the present study.	3
